# Colloidal analogues of polymer chains, ribbons and 2D crystals employing orientations and interactions of nano-rods dispersed in a nematic liquid crystal

**DOI:** 10.1038/s41598-019-40198-1

**Published:** 2019-03-15

**Authors:** Muhammed Rasi M, Ravi Kumar Pujala, Surajit Dhara

**Affiliations:** 1 School of Physics, University of Hyderabad, Hyderabad, 500046 India; 2 Present Address: Indian Institute of Science Education and Research, Tirupati, India

## Abstract

Robust control over the position, orientation and self-assembly of nonspherical colloids facilitate the creation of new materials with complex architecture that are important from technological and fundamental perspectives. We study orientation, elastic interaction and co-assembly of surface functionalized silica nano-rods in thin films of nematic liquid crystal. With homeotropic boundary condition, the nano-rods are predominantly oriented perpendicular to the nematic director which is different than the mostly parallel orientation of the micro-rods. The percentage of perpendicular nano-rods are significantly larger than the parallel nano-rods. The perpendicular nano-rods create very weak elastic deformation and exhibit unusual, out-of-plane, attractive interaction. On the other hand, the nano-rods oriented parallel to the director create strong elastic deformation and shows anisotropic, in-plane, dipolar interaction. In both orientations, the induced defects reside in the nano-rods. With the help of a dynamic laser tweezers and using nano-rods as building blocks we demonstrate colloidal analogues of linear polymer chains, ribbons and two-dimensional binary crystals.

## Introduction

Achieving colloidal analogues of atoms, molecules and chemical elements has been challenging in colloidal science. In this field, significant advances have been made in variety of systems such as janus magnetic rods, DNA coated patchy colloids, co-assembly of soft patchy nanoparticles, paramagnetic colloids, photocatalic colloids etc^1–9^. Mostly they are dispersed in an isotropic medium like water and the interaction forces are short-range. When colloidal particles are dispersed in nematic liquid crystals they create strong elastic deformation in the director field denoted by a dimensionless unit vector, **n**^10^. The deformation often culminates in the form of topological defects in the vicinity of the colloids depending on the surface anchoring and the shape of the colloids^11–16^. The colloids interact anisotropically via long-range elastic forces which have no analogues in regular colloidal systems in an isotropic dispersive medium. The elastic forces in liquid crystal are usually dipolar or quadrupolar type akin to electrostatics. The interplay of defect and elastic interaction gives rise to complex colloidal structures and superstructures starting from one to three-dimensions^17–21^. The diversity in the structure is often introduced by varying the shape, size and genus of the colloids^17,22–29^.

In this paper, we report experimental studies on the dispersion of silica nano-rods in a nematic liquid crystal. We focus on the spontaneous orientation, elastic interaction and directed co-assembly with micro-rods and microspheres in a nematic liquid crystal. We find that the silica nano-rods are predominantly oriented orthogonal to the nematic director and exhibit out-of-plane attractive interaction. The induced defects reside in the nano-rods. Using dynamic laser tweezers we have designed various colloidal analogues of linear polymer chains, ribbons and two dimensional binary colloidal crystals. Ours is the first experiment among several nematic colloidal systems, showing spontaneous out-of-plane interaction giving rise to vertical assembly of silica nano-rods in a liquid crystal. Such studies have important bearing on the efforts in making three-dimensional, periodic nematic colloidal systems, in which the orientational direction of the nano-rods is orthogonal to the nematic director.

## Results and Discussion

**Figure 1:**
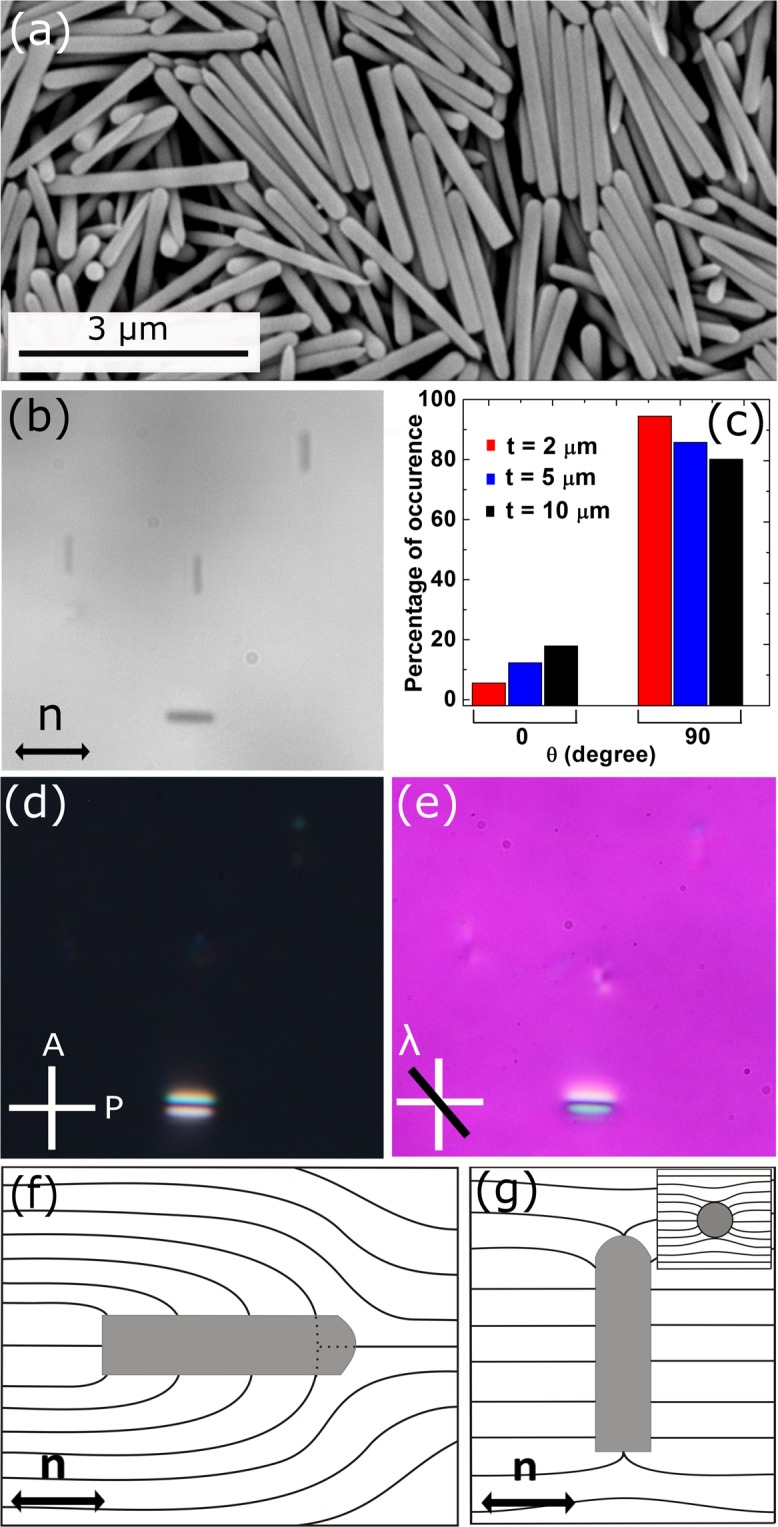
(**a**) SEM image of silica rods of mean length and diameter are *l* = 3 μm and *d* = 200 nm respectively (designated as nano-rods). (**b**) CCD image of a few DMOAP coated silica nano-rods dispersed in a planar cell of 5CB liquid crystal, (**c**) Statistics of orientation of the nano-rods with respect to the director. Different colours correspond to different cell thicknesses namely, 2, 5 and 10 μm respectively. POM images with, (**d**) crossed polarisers, (**e**) an additional *λ*-plate (530 nm) inserted between the polarisers and sample. Schematic diagram of director distortions surrounding a nano-rod oriented, (**f**) parallel to the director, and (**g**) perpendicular to the director. (Inset) Cross-section of director distortion. Double headed arrow below **n** denotes the director. White cross denotes polariser and analyser.

Our study begins with the observation of orientation of DMOAP coated silica nano-rods dispersed in a planar cell of 5CB liquid crystal. The length to diameter aspect ratio (*l*/*d*) of these nano-rods is about 15. Figure 1(b) shows a representative CCD image of a few dispersed nano-rods. It is observed that the nano-rods are oriented either parallel or perpendicular to the director (**n**). The optical micrographs with cross polarisers (POM) and with an additional *λ*-plate are shown in Fig. 1(d,e) respectively. The parallel nano-rods are seen clearly due to strong elastic distortion whereas the perpendicular nano-rods are obscured. The yellow and blue colours around the parallel nano-rod in Fig. 1(e) represents anticlockwise and clockwise rotation of the director, respectively. Analysing the *λ*-plate image we present schematic director deformation for both orientations of a nano-rod. The point defect in case of parallel orientation (Fig. 1(f)) resides in the nano-rod and the ring defect along the perpendicular nano-rod (Fig. 1(g)) is highly pinned on the surface and hence unresolvable. The cross section of a perpendicular nano-rod in the inset of Fig. 1(g) shows that the elastic distortion is very weak.

**Figure 2:**
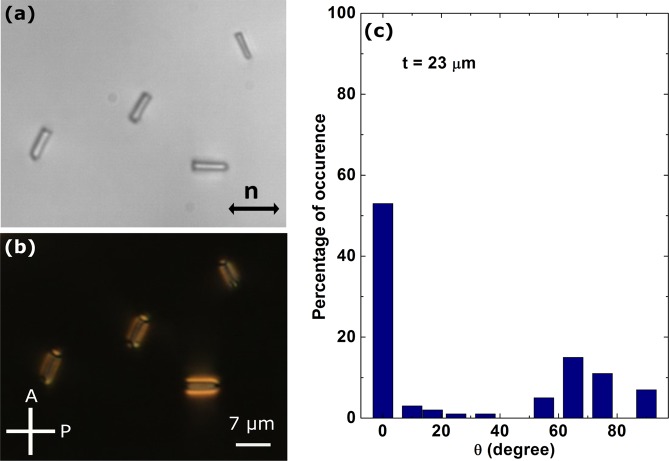
Polarising optical microscopy (POM) images of silica rods of average length 6.5 μm and diameter 0.75 μm (designated as micro-rods) dispersed in a planar cell (5CB), (**a**) without crossed polarisers, (**b**) with crossed polarisers. (**c**) Statistics of orientation with respect to the director (**n**). Cell thickness is 23 μm.

To find the size effect we studied the orientation of silica rods with larger length and diameter than the nano-rods. Transmission electron microscope image of the micro-rods and Movies are available in the Supplementary Materials (Fig. S). The mean length and diameter are about 6.5 μm and 0.75 μm respectively. They are designated here as micro-rods. The aspect ratio of these micro-rods is: *l*/*d* ≃ 9, i.e., almost half the value of the nano-rods. Figure 2(a) shows a CCD image of a few DMOAP coated micro-rods dispersed in 5CB liquid crystal. It is observed that majority of the micro-rods are orientated parallel to the director and minority of them orientated unevenly in all other directions (Fig. 2(a,b)). This is in sharp contrast to the orientation of the nano-rods just discussed (see Fig. 1(b)). The induced defects by the micro-bullet particles with one blunt end and one hemispherical end have been studied in nematic liquid crystals^14^. The length and diameter of the particles were 10 μm and 2 μm respectively and the aspect ratio is almost half of our micro-rods. They reported that micro-bullets with homeotropic surface anchoring form elastic dipoles with a nematic point defect located near the curved end and are aligned along the director. In our micro-rod system we get elastic dipoles but the the point defects are found to form on either side. This means the effect of edge asymmetry is negligible. We obtained statistics of the orientation of both the nano-rods and micro-rods. About 150 nano-rods and 120 micro-rods were studied for this purpose. Figure 1(c) shows that nano-rods have two distinct orientations with majority (more than 80%) of them are perpendicular to the director. The percentage of occurrence weakly depends on the cell thickness for the nano-rods. In contrast, majority of the micro-rods (more than 50%) are oriented parallel to the director and the distribution is wide (Fig. 2(c)). Comparatively in thinner cells the number of parallel nano-rods is larger (Fig. 1(c)). The statistics and elastic deformation of our micro-rods are almost similar to that of the glass micro-rods studied by Tkalec *et al*.^19^.

The difference in the orientation behaviour of the silica nano and micro-rods can be understood based on a dimensionless parameter^30,31^. Assuming negligibly small contribution of the edges, the elastic energy and the surface energy of the silica rods can be written as *E*_*el*_ ≈ *LK*, and *E*_*sur*_ ≈ *RLW*, where *L* is the length, *R* the radius and *K* is the average elastic constant and *W* is the anchoring energy coefficient. The ratio of these two energies gives a dimensionless parameter, which is written as: *p* = *WR*/*K*. If *p* > 1, the silica rods are oriented parallel and if *p* < 1 they are oriented perpendicular to the director. For a given *W*, the orientation mainly depends on the radius of the silica rod as *p* ∝ *R*. The diameter of the nano-rods is almost 4 times smaller than that of the micro-rods, hence *p* could be less than one and the perpendicular orientation of the majority of nano-rods is favourable. Also it has been shown that for nano-rods with high aspect ratio the surface anchoring energy per rod can be written as *F* = (*πLDW*)(1 + *cos*^2^*θ*)/4, where *D* is the diameter and *θ* is the angle between the director and the long axis of the nano-rod. This energy is minimised when they are perpendicular to the director i.e., *θ* = ±*π*/2^17^.

**Figure 3:**
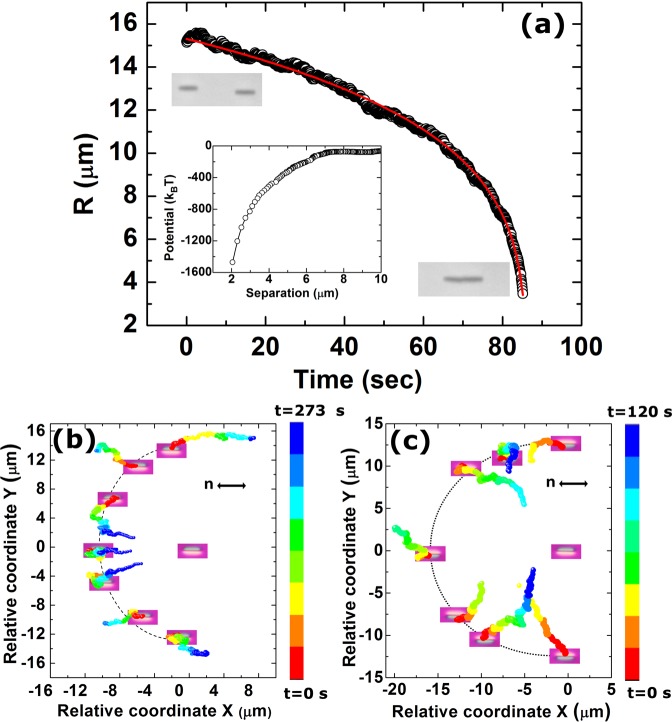
(**a**) Time dependent centre to centre separation (*R*) between a pair of collinear nano-rods parallel to the director (Movie S1)^30^. Red line shows the nonlinear least square fit to $R(t)=({R}_{0}^{5}-5\alpha t{)}^{\mathrm{1/5}}$ with *α* =7.3 × 10^3^μm^5^/sec corresponding to dipolar interaction. Inset shows the interaction potential as a function of separation. Colour coded time trajectories (Relative coordinate) of two nano-rods oriented parallel to the director and situated at different angles with respect to the director when both the nano-rods having, (**b**) similar director orientation, (**c**) opposite director orientation (see the yellow and blue colours of the *λ*-plate images in two cases).

In what follows, we study elastic pair interaction of nano-rods oriented both parallel and perpendicular to the director. Figure 3 presents the result of two interacting nano-rods oriented parallel to the director. Two nano-rods were kept apart at a distance with the help of the laser tweezers and allowed to interact freely. The centre to centre separation (*R*) was measured as a function of time and shown in Fig. 3(a). The time dependent separation corresponding to dipolar interaction is fitted to the equation^32^: $R(t)={({R}_{0}^{5}-5\alpha t)}^{1/5}$, where *α* = *k*/*ζ*, *ζ* being the drag coefficient and *R*_0_ in the separation at *t* = 0. This confirms that the elastic interaction is dipolar type and the corresponding variation of potential energy is shown in the inset. Anisotropy of the interaction was studied by measuring the relative coordinates of two nano-rods approaching each other from different angles (Fig. 3(b,c)). Trajectories inside and outside the dotted semicircles represent attractive and repulsive interaction respectively. When two nano-rods have similar elastic distortions (both have same colour layout with *λ*-plate) they have attractive interaction within a narrow angle of about ±15°, and repulsive at all other angles (Fig. 3(b)). When they are colinear and have opposite elastic deformation (opposite layout of colour with respect to the previous pair) the interaction is repulsive and attractive for all other angles (Fig. 3(c)).

**Figure 4:**
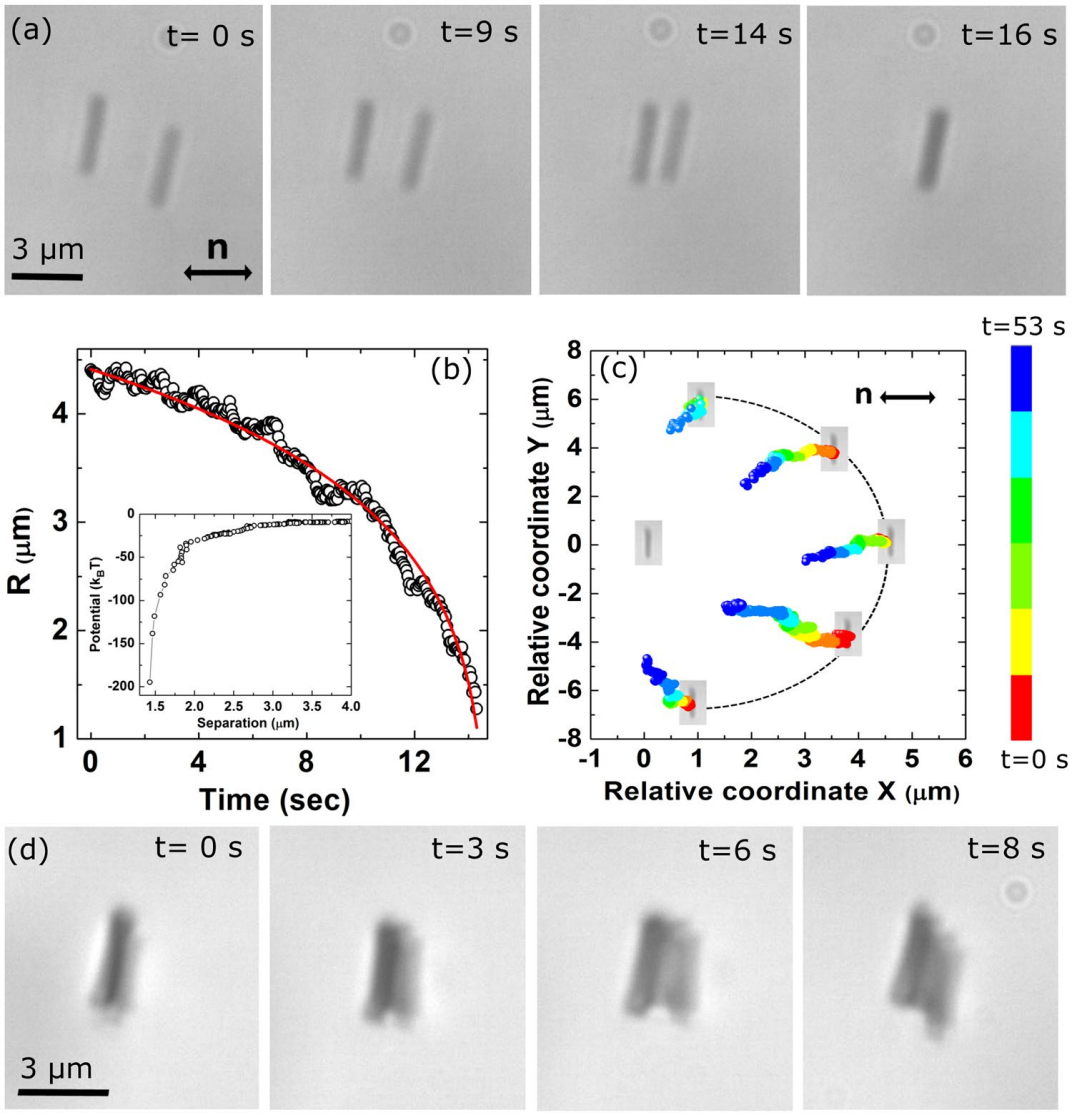
(**a**) Sequence of CCD images of a pair of interacting nano-rods oriented perpendicular to the director. Nano-rods are attracted towards each other and one nano-rod goes below the other (Movie S2). (**b**) Time dependent centre to centre separation (*R*) of a pair of nano-rods. The red line shows nonlinear least square fit to $R(t)=({R}_{0}^{7}-7\alpha t{)}^{\mathrm{1/7}}$ with *α* = 2.4×10^4^ μm^7^/sec corresponding to quadrupolar interaction. Inset shows the variation of interaction potential with separation. (**c**) Colour coded time trajectory (Relative coordinate) of two nano-rods approaching each other at different angles with respect to the director. (**d**) Vertical ribbon-like structure made of fourteen nano-rods and temporal evolution of the structure when a gentle force was applied with the help of the optical tweezers.

We study the elastic pair interaction of nano-rods oriented perpendicular to the director. A few representative CCD images taken at different times of two interacting nano-rods are shown in Fig. 4(a). It is observed that due to the attractive interaction one nano-rod goes under the other in the vertical direction (out-of-plane). The variation of interparticle separation (Fig. 4(b)) can be fitted to the equation^32^; $R(t)={({R}_{0}^{7}-7\alpha t)}^{\mathrm{1/7}}$, corresponding to the quadrupolar interaction. The variation of interaction potential is also shown in the inset. The relative coordinates of two interacting nano-rods show that the attractive interaction is short-range and does not depend on the approaching angle hence the interaction is isotropic (see Fig. 4(c)). A vertical ribbon-like structure is prepared by assembling 14 nano-rods and shown in Fig. 4(d). All the nano-rods can not be seen from the top because of vertical assembly. To visualise the ribbon properly and check the stability, the structure was tilted by dragging with the help of the laser tweezers. Simultaneously images were captured at different time intervals as shown in the subsequent images. Finally the structure relaxes back to the initial configuration when the laser is switched off. The interaction of spherical nanoparticles dispersed in nematic liquid crystals has been studied by Ryzhkova *et al*.^24^, and found to be always in the plane of the sample. Thus out-of-plane attractive isotropic interaction of nano-rods is unusual and potential for making hierarchical materials assembly.

**Figure 5:**
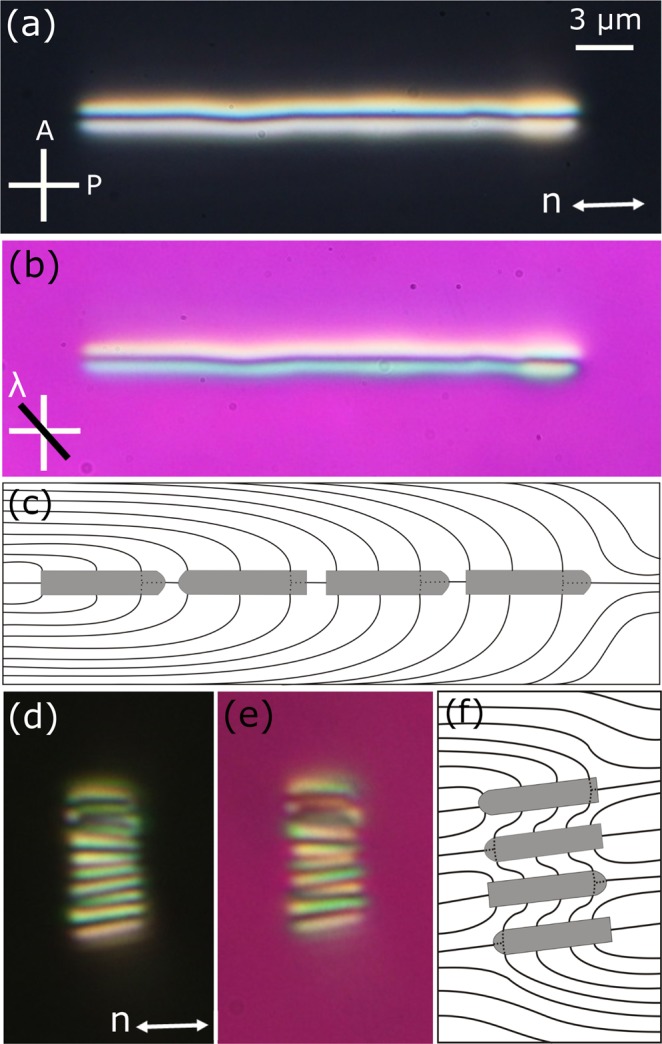
POM images of a 1D linear chain of 10 nano-rods assembled along the nematic director. Images are taken with, (**a**) crossed polarisers, (**b**) an additional *λ*-plate. (**c**) Schematic representation of the director field surrounding the chain. (**d**,**e**) POM and red-plate images of a ribbon-like structure assembled in the perpendicular direction of the director. (**f**) Schematic director orientation surrounding the ribbon.

We construct a variety of stable and linear colloidal structures employing the nano-rods. Figure 5(a,b) shows a long chain of ten nano-rods, which are oriented along the director. It appears as a continuous string like object without any discontinuity in-between. The linear chain of nano-rods is akin to a linear homopolymer in which each nano-rod resembles a monomer. A schematic director orientation surrounding the chain is shown in Fig. 5(c). In the similar way ten nano-rods were assembled side-by-side to form a horizontal ribbon-like structure as shown in Fig. 5(d,e). A schematic director orientation surrounding the ribbon is also shown in Fig. 5(f).

**Figure 6:**
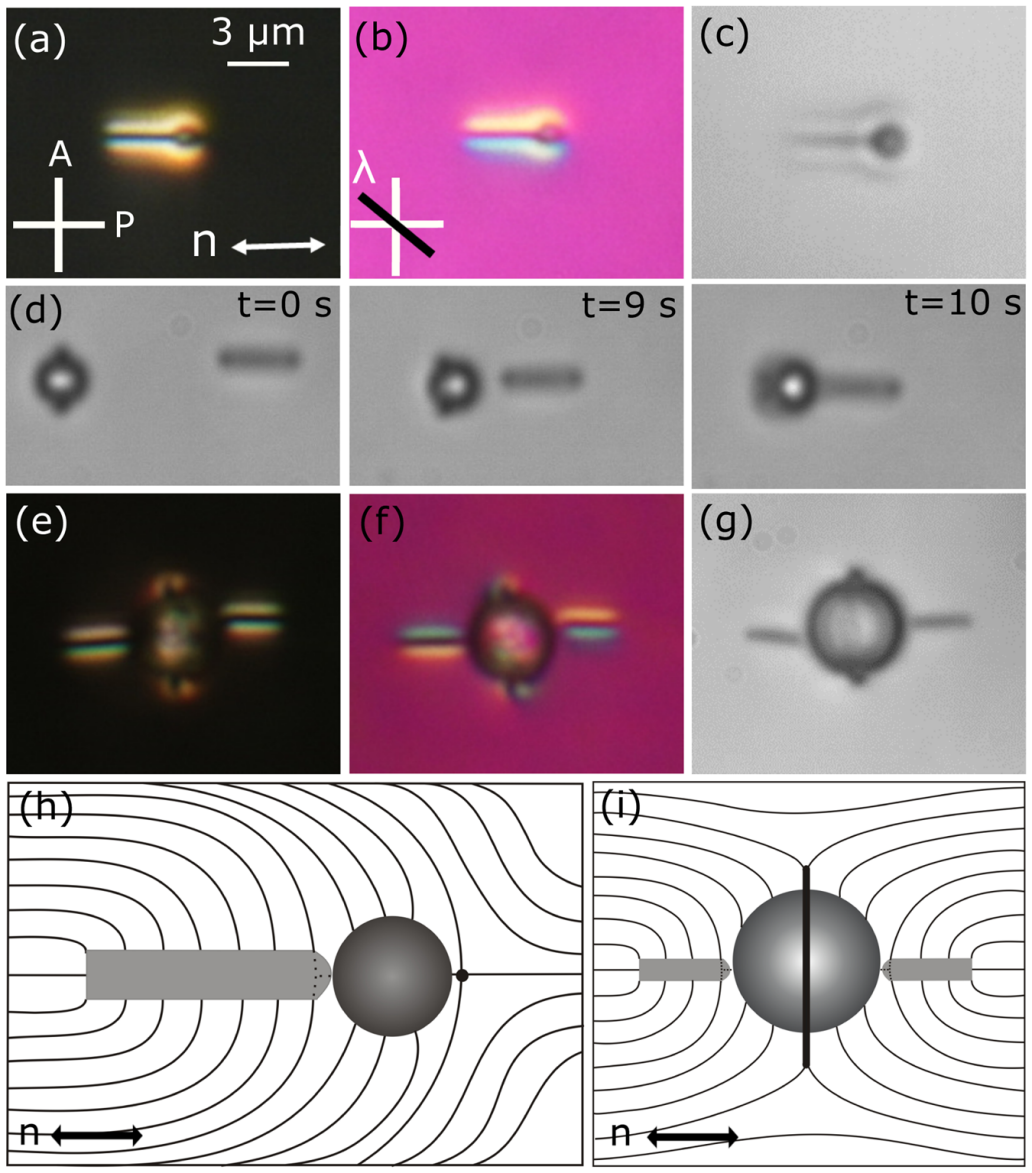
Match-stick like shape formed by a dipolar spherical colloid of diameter 1.1 μm and a nano-rod. POM images with, (**a**) cross polarisers, (**b**) an additional *λ*-plate, and (**c**) without polarisers. (**h**) Schematic representation of director deformation surrounding the assembly of the nano-rod and microsphere. (**d**) Temporal evolution of conversion of a spherical quadrupolar colloid of diameter 2.3 μm into dipolar one when the nano-rod and the microsphere are approaching each other (Movie S3). (**e**–**g**) Assembly of a quadrupolar colloid of diameter 5.2 μm and two nano-rods oriented opposite sides and (**i**) schematic director deformation.

To construct co-assembled structures we used DMOAP coated microspheres and nano-rods. A dipolar microsphere of diameter 1 μm and a nano-rod oriented parallel to the director have attractive interaction and forms a match-stick like object (Fig. 6(a–c)). This helps to understand in which side of the nano-rod the point defect is hidden. For example, the *λ*-plate image ((Fig. 6(b)) clearly suggests that the point defect is residing close to the right side of the nano-rod. When a quadrupolar microsphere is allowed to interact with the nano-rod, the result is very different. In this case, the Satrun ring defect of the microsphere becomes unstable as they approach to each other and finally the quadrupolar microsphere is converted to a dipolar microsphere. A sequence of CCD images taken at different times of a 2.3 μm microsphere are shown in Fig. 6(d). The experiments were performed with varying diameter of the microspheres and found that beyond about 3.5 μm microsphere, the quadrupolar structure is stable and no transformation from quadrupole to dipolar is observed. For example, Fig. 6(e–g) shows a stable association when two nano-rods from the opposite directions were allowed to interact in the equatorial plane of a spherical colloid of diameter 5.2 μm. The interaction is attractive and the assembly as a whole looks like a snake charmer’s flute. If they approach from any other plane then they are attracted to the Saturn ring similar to the response of spherical nanoparticles^24^.

**Figure 7:**
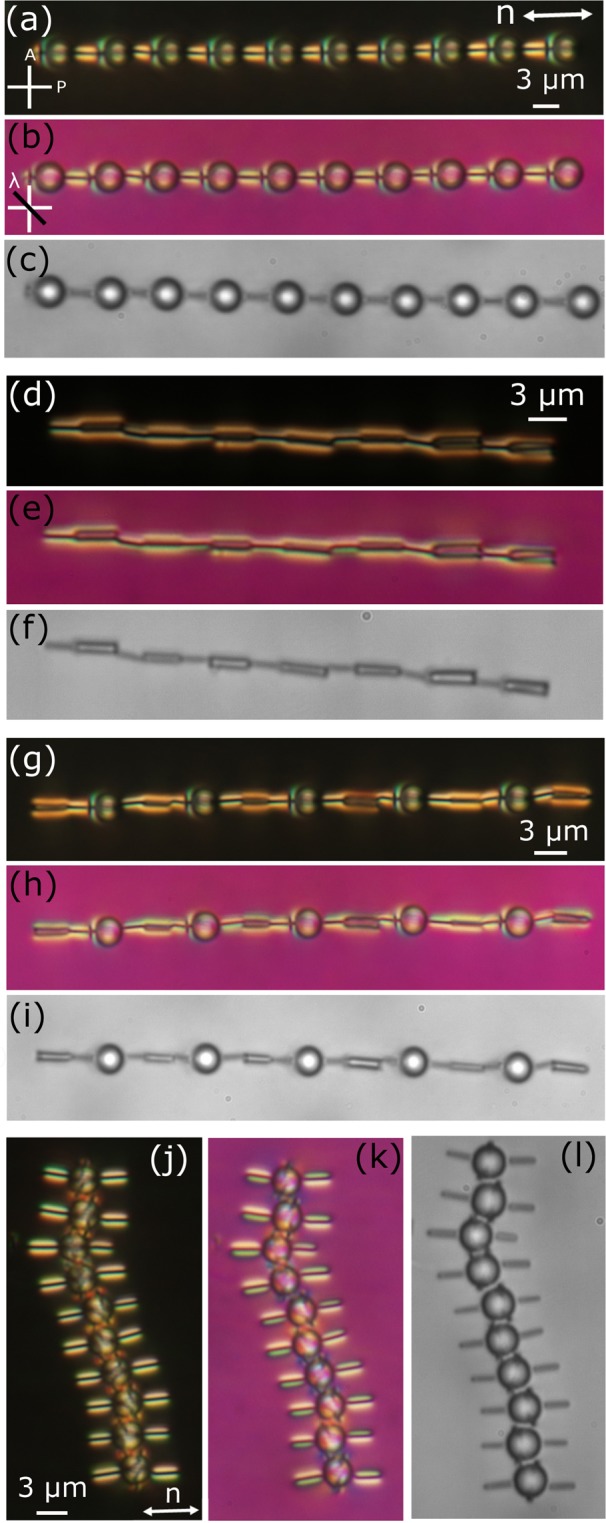
POM images of linear chains of colloidal co-assembly of microspheres, micro-rods and nano-rods. Images are taken with (**a**,**d**,**g**,**j**) crossed polarisers, (**b**,**e**,**h**,**k**) an addditional *λ*-plate, and (**c**,**f**,**i**,**l**) without cross polarisers. The diameter of the microsphere is 5.2 μm.

Using different combination of nano-rods, micro-rods and microspheres, we prepared several linear chains as shown in Fig. 7. Figure 7(a) shows the optical photomicrograph of a linear chain of assembly of several 5.2 μm diameter dipolar colloids and nano-rods. The binding energy for pair interaction is approximately 4000 k_B_T. The colloids were arranged in an alternative fashion and the linear chain is parallel to the director. Corresponding images taken with *λ*-plate and without cross polarisers are also shown in Fig. 7(b,c). The surface to surface separation between the two microspheres is almost equal to the length of the nano-rods as the induced defects of the nano-rods are engulfed. Similar way, we prepared linear chains of alternating micro-rods and nano-rods (Fig. 7(d–f)) and micro-rods, nano-rods followed by microspheres (Fig. 7(g–i)). The binding energy for pair interaction of nano and micro-rods is approximately 3000 k_B_T. We also prepared a linear chain of quadrupolar microspheres and nano-rods. In this case, the chain is orientated perpendicular to the director. The binding energy for pair interaction is approximately 1500 k_B_T and comparatively weaker than the previous two cases. These structures are akin to linear copolymer chains. For example, the structure shown in Fig. 7(a,d) can be considered as a linear copolymer of (-A-B-)_n_ type. The structure shown in Fig. 7(g) is akin to a copolymer of (-A-B-C-B-)_n_ type. The linear chain shown in Fig. 5(a) is analogous to a homopolymer of (-A-A-)_n_ type. The polymeric structures are highly stable and reconfigurable by the optical tweezers.

**Figure 8:**
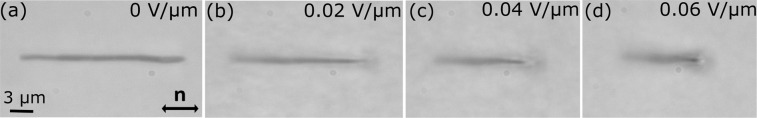
CCD images of a linear chain of 7 nano-rods in a planar cell showing the effect of ac electric field. Two ends of the chain get defocused as it tends to align along the field direction. Frequency of the field: 20 kHz. Cell thickness: 50 μm. See video in the supplementary material (Movie S4).

The robustness of the colloidal polymers is tested by applying electric field. Figure 8 shows the effect of ac field perpendicular the chain and the director. With increasing electric field, the chain reorients toward the field direction beyond the Freedericksz’s threshold field since the dielectric anisotropy of 5CB is positive. The reorientation is reversible without creating any deformation in the structure (see SI).

**Figure 9:**
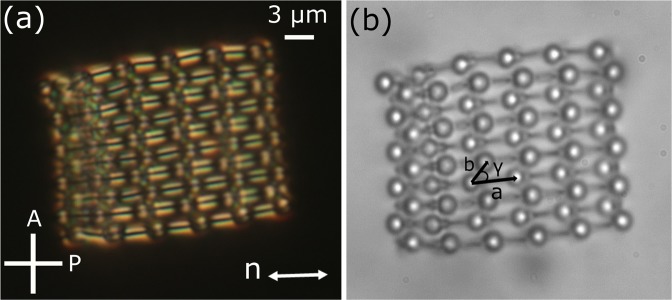
Optical photomicrographs of a 2D crystal of binary colloids. POM images taken, (**a**) with and (**b**) without cross polarisers. Oblique lattice with parameters; *a* ≃ 7.2 μm, *b* ≃ 3.9 μm and *γ* ≃ 48°.

As a next step, the colloidal copolymer chains are assembled by using the laser tweezers for making two dimensional binary crystal. Figure 9 shows a two-dimensional binary colloidal crystal prepared by assembling (-A-B-)_n_ type copolymer chains of nano-rods and microspheres in a planar cell. As a result of long-range elastic interaction a stable 2D oblique lattices are assembled. The diameter of the microspheres and the length of the nano-rods are same i.e., 3 μm. Two colloidal copolymer chains oriented in an antiparallel way and assembled with the help of the optical tweezers. There are 60 dipolar microspheres and 50 nano-rods in the structure shown in Fig. 9(b)). The lattice unit is generally a parallelogram with average lattice parameters *a* = 7.2 μm, *b* = 3.9 μm and angle $\gamma \simeq {48}^{\circ }$. It appears that some microspheres are brighter than others. This means all the colloids are not in one plane. However, the structure is highly stable. More complex colloidal structures can be prepared by designing appropriate polymer as base unit and could be useful for photonic applications.

## Conclusion

The nano-rods orient very differently than the micro-rods in nematic liquid crystals. The nano-rods exhibit only two specific orientations, namely the parallel or perpendicular, with respect to the nematic director. Majority of them are oriented perpendicular to the nematic director. The induced defects are virtual and hence unresolvable under microscope. On the other hand, micro-rods show all possible orientations unevenly with 50% of them oriented parallel to the director. They are either elastic dipolar or quadrupolar micro-rods. Nano-rods oriented perpendicular to the director experience out-of-plane isotropic attractive quadrupolar interaction in contrast to the in-plane anisotropic interaction of the nano-rods oriented parallel to the director. Smaller spherical quadrupolar colloids are unstable and transformed into a dipolar type in the presence of nano-rods. Colloidal chains akin to linear copolymers such as (-A-B-)_n_, (-A-B-C-B-)_n_, homopolymer (-A-A-)_n_, horizontal and vertical ribbons, 2D binary crystals are designed by choosing variety of colloids and directing their assembly by laser tweezers. Polymeric structures are robust as they can be oriented along the applied electric field reversibly without any permanent deformation. Orthogonal orientation of the nano-rods with respect to the nematic director is potential for making hybrid molecular-colloidal liquid crystal system in which the direction of colloidal ordering is orthogonal to the nematic director. The spontaneous vertical assembly of the nano-rods could provide a unique and versatile route toward building blocks for hierarchical materials assembly.

## Materials and Methods

The silica rods were synthesised by a wet chemical method and prepared following the procedure of Kujik *et al.*^33^. First, 3 g of PVP (Polyvinylpyrrolidone) was dissolved in 30 ml of 1-pentanol. After complete dissolution of PVP, 3 ml of ethanol (100%, Interchema), 0.84 ml Milli Q water and 0.2 ml aqueous sodium citrate dyhydrate (0.17 M, 99% Sigma-Aldrich) were added. The mixture was shaken thoroughly and made bubble free. Finally, 0.3 ml of Tetraethyl orthosilicate (TEOS, 98% Sigma-Aldrich) was added to the reaction mixture, shaken gently and the reaction was left to continue for 24 h. The mixture was then centrifuged and fractionated to obtain colloids with desired aspect ratio. The average length (*l*) and diameter (*d*) of the silica rods are; *l* = 3 μm and *d* = 200 nm respectively (designated as nano-rods). We also prepared longer and thicker silica rods of length and diameter; *l* = 6.5 μm and *d* = 0.75 μm respectively (designated as micro-rods). Silica microspheres used in the experiment were obtained from Bangs Chemicals (USA). The scanning electron microscope (SEM) image of the synthesised silica nano-rods is shown in Fig. 1(a). One end of the nano-rods is flat and the other end is hemispherical. They were coated with DMOAP (Octadecyldimethyl-3-trimethoxysilyl propyl-ammonium chloride) that gives homeotropic anchoring of the nematic director and dispersed in 5CB (4-n-pentyl-4-cyanobiphenyl) liquid crystal. The colloidal mixture was introduced in planar cells coated with polyimide AL-1254. A dynamic laser tweezer was built around an inverted optical polarising microscope (Nikon Eclipse Ti-U) using a cw solid-state laser operating at 1064 nm^32,34–39^. An acousto-optic deflector (Aresis, Tweez 250Si) interfaced with a computer was used for trap movement. The position and orientation of the nano and micro-rods were tracked by using appropriate software with a resolution of ±10 nm.

## Supplementary information

Movie-S1

Movie-S2

Movie-S3

Movie-S4

Supplementary Materials
