# Supplementary material for: Colloidal analogues of polymer chains, ribbons and 2D crystals employing orientations and interactions of nano-rods dispersed in a nematic liquid crystal
Source: Sci Rep. 2019 Mar 15;9:4652. doi: 10.1038/s41598-019-40198-1 (PMC6420569; doi:10.1038/s41598-019-40198-1)
Supplement: Supplementary file 5 — Supplementary Materials [file 41598_2019_40198_MOESM5_ESM.pdf]

Supplementary Materials

Colloidal analogues of polymer chains, ribbons and 2D crystals employing orientations and interactions of nano-rods dispersed in a nematic liquid crystal

Muhammed Rasi M, Ravi Kumar Pujala, and Surajit Dhara*
School of Physics, University of Hyderabad, Hyderabad-500046, India

*Email:sdsp@uohyd.ernet.in

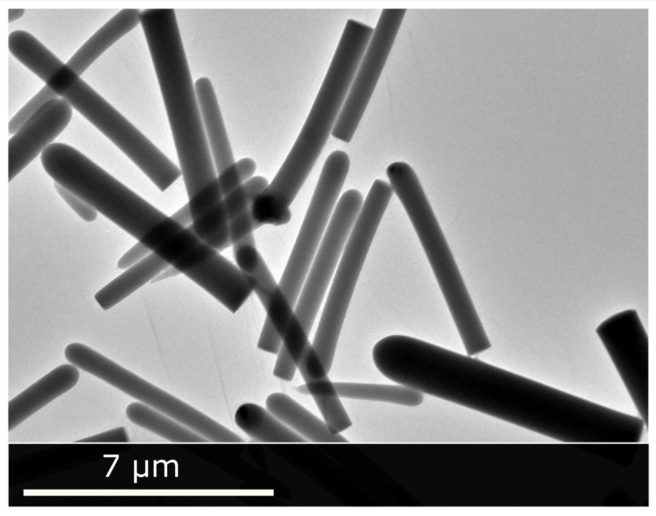

Fig.1: Transmission electron microscope image of the micro-rods of average length 6.5μm and diameter 0.75 μm respectively.

Supplementary movies

Movie-S1(File name: S1.avi)

Collinear interaction of a pair of DMOAP coated silica nano-rods of diameter 200nm length 3.5μm orientated parallel to the director in a planar cell of 5CB liquid crystal.

Movie-S2 (File name: S2.avi)

Out of plane interaction of a pair of DMOAP coated silica nano-rods of diameter 200nm length 3.5 μm oriented perpendicular to the director in a planar cell.

Movie-S3 (File name: S3.avi)

Interaction between a DMOAP silica nano-rod and a quadrupolar microsphere of diameter 2.3 μm.

Movie-S4 (File name: S4.avi)

Effect of increasing and decreasing electric field on a linear chain of nano-rods in a planar cell of 5CB. The linear chain is consists of 7 nano-rods aligned parallel to the director. The field is slowly increased beyond Freedericksz's threshold field and then decreased. Cell thickness 50 μm.
